# Insights Into the Proteomic Profiling of Extracellular Vesicles for the Identification of Early Biomarkers of Neurodegeneration

**DOI:** 10.3389/fneur.2020.580030

**Published:** 2020-12-11

**Authors:** Ricardo Quiroz-Baez, Karina Hernández-Ortega, Eduardo Martínez-Martínez

**Affiliations:** ^1^Departamento de Investigación Básica, Dirección de Investigación, Instituto Nacional de Geriatría, Ciudad de México, Mexico; ^2^Departamento de Genética del Desarrollo y Fisiología Molecular, Instituto de Biotecnología, Universidad Nacional Autónoma de México, Cuernavaca, Mexico; ^3^Laboratory of Cell Communication & Extracellular Vesicles, Division of Basic Science, Instituto Nacional de Medicina Genómica, Ciudad de México, Mexico

**Keywords:** extracellular vesciles, central nervous system, neurodegenenerative diseases, biomarkers, proteomic analyses, Parkinson' s disease, Alzheimer's disease, endolysosomal dysfunction

## Abstract

Extracellular vesicles (EVs) are involved in the development and progression of neurodegenerative diseases, including Alzheimer's and Parkinson's disease. Moreover, EVs have the capacity to modify the physiology of neuronal circuits by transferring proteins, RNA, lipids, and metabolites. The proteomic characterization of EVs (exosomes and microvesicles) from preclinical models and patient samples has the potential to reveal new proteins and molecular networks that affect the normal physiology prior to the appearance of traditional biomarkers of neurodegeneration. Noteworthy, many of the genetic risks associated to the development of Alzheimer's and Parkinson's disease affect the crosstalk between mitochondria, endosomes, and lysosomes. Recent research has focused on determining the role of endolysosomal trafficking in the onset of neurodegenerative diseases. Proteomic studies indicate an alteration of biogenesis and molecular content of EVs as a result of endolysosomal and autophagic dysfunction. In this review, we discuss the status of EV proteomic characterization and their usefulness in discovering new biomarkers for the differential diagnosis of neurodegenerative diseases. Despite the challenges related to the failure to follow a standard isolation protocol and their implementation for a clinical setting, the analysis of EV proteomes has revealed the presence of key proteins with post-translational modifications that can be measured in peripheral fluids.

## Introduction

Neurodegenerative diseases (NDs) are complex disorders with devastating consequences for the patient and their immediate social environment. In Alzheimer's disease (AD) and Parkinson's disease (PD), the neuronal dysfunction and secondary effects progress gradually with heterogeneous clinical outcomes. Despite the advances in management of AD and PD, these diseases are among the leading causes of disabilities and showed an increased burden especially in low- and middle-income countries (1). One of the major challenges in the field of neuroscience is to achieve an early and precise detection of NDs to procure an adequate treatment and delay their progression. Most of the patients are diagnosed when overt symptoms of neurodegeneration are displayed. Furthermore, it has been documented that the misdiagnosis rate of AD and PD could be as high as 17 and 25%, respectively (2–5). Thus, there is a need to develop new diagnostic strategies to aid in the identification of people developing AD and PD.

The specific cause of neural cell death has not been resolved, but it has become clearer that in AD and PD, both genetic and environmental factors operate. In the last two decades, several chromosomal regions have been identified containing genes that are associated with the risk of developing these NDs. In the early-onset variety of AD and PD, a point mutation could be identified as a causal agent due to the impairment of the function of a specific protein. Among the most relevant genes that contain genetic variants for AD development are the *APP, PSEN1, PSEN2, APOE, ADAM10*, and *ACE* genes (6). In the case of PD, the development of the disease has been linked to the following genes: *SNCA, PRKN, PINK1, DJ-1, LRRK2, ATP13A2, PLA2G6, FBX07*, and *VPS35* (7, 8). Despite early-onset cases representing <10% of the total AD and PD cases, these studies have helped to determine the main processes that underlie neurodegeneration processes. In general, these two diseases are characterized by alterations in mitochondrial function, in management of oxidative stress, in protein folding and aggregation, and in immune function (7–9).

The diversity and severity of symptoms and the differences in the timing of neurodegenerative progression have made it difficult to create clinically applicable tests for the diagnosis of AD and PD. In many cases, the confirmation of a ND is performed post-mortem. For this reason, the definition of a set of biomarkers is essential for diagnosis, stratification of patients and therapeutic monitoring. In recent years, the fact that altered protein homeostasis is a common event in the development of NDs has guided the development of diagnostic methods. For instance, several studies have found that decreased levels of amyloid β peptide of 42 amino acids length (Aβ42) in AD and of α-synuclein (α-syn) in PD in cerebrospinal fluid (CSF) indicate an overt development of the disease, but low specificity and contradictory results have been reported (10, 11). Nevertheless, these findings set the precedent for the identification of circulant biomarkers indicating the progress of NDs. A relevant aspect in the development of AD and PD is the dysfunction of the interaction between endosomes, lysosomes, and mitochondria at various levels. These alterations modify the secretory capacities of the cells in the brain, which is reflected in the molecular composition of organelles released into the extracellular space, which are known as extracellular vesicles (EVs).

**Figure 1:**
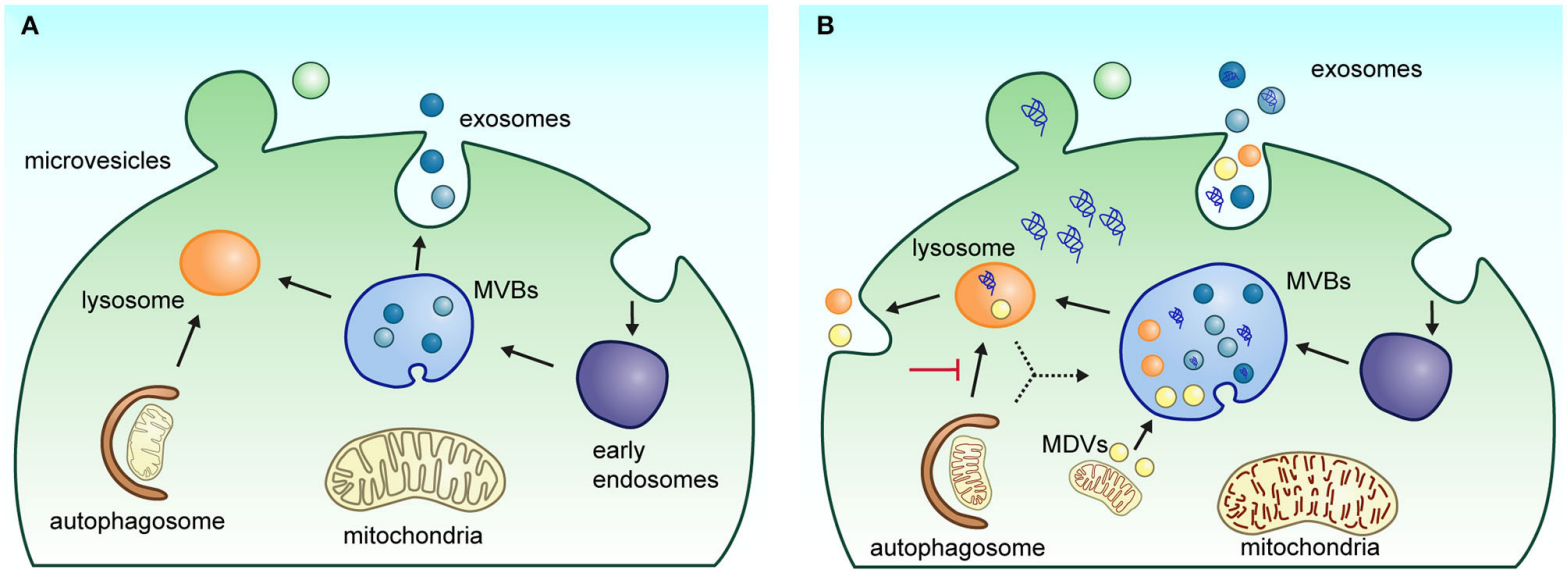
Biogenesis and secretion pathway of EVs. **(A)** Under physiological conditions, extracellular vesicle biogenesis and secretion process occur in two main ways: (1) exosomes are generated in multivesicular bodies (MVBs) following the endolysosomal pathway; when MVBs fuse with the plasma membrane, exosomes are released into the extracellular space; or (2) microvesicles are released directly by budding from the plasma membrane. **(B)** Under neuropathological conditions such as Parkinson's and Alzheimer's disease, alterations in the endolysosomal system can affect biogenesis and release of EVs in the brain cells. The impairment of the autophagic–lysosomal pathway and alteration in protein sorting contribute to the increased accumulation of pathological markers in EVs. Several neurological diseases show an enlargement of MVBs with an increased number of intraluminal vesicles. The deficient mitophagy process also induces dysfunctional mitochondria accumulation, favoring the generation and release of mitochondrial-derived vesicles (MDVs) by cells.

The generic term of EVs refers to the lipidic vesicles that are released from the endosomal system (exosomes) and from the plasma membrane (microvesicles or ectosomes) (Figure 1A). EVs are considered a novel system of intercellular communication that involves the transfer of proteins, RNA, lipids, and metabolites. An alteration of the molecular cargo of EVs has been associated with the development of several diseases. In the case of NDs, it has been proposed that these organelles are involved in the propagation of mis-folded proteins in the brain (12). Moreover, it has also been documented that part of the communication between glia and neurons is mediated by EVs and their alteration has implications for the development of NDs. The advantage of studying EVs is that exosomes and microvesicles can be isolated from fluids such as CSF, blood, saliva, or urine. Thus, the molecular characterization of EVs is promising because the analysis in both patient samples and disease models may reflect the type of molecules that are expressed at different stages of the development of NDs. Interestingly, it has been described that brain-derived exosomes can be found in peripheral circulation. In this review, we will focus on discussing the scope and limitations of the proteomic characterization of EVs and their potential to identify molecules that guide the development of non-invasive tests and to help unravel neurodegenerative pathophysiology.

## Role of EVs in the Central Nervous System

The intercellular communication mediated by EVs has been linked to various processes of normal brain function both in development and in adulthood. During the synaptogenesis process and proliferation of neural progenitors, the gradients of hydrophobic molecules such as Wnt and Sonic the Hedgehog are generated by their release and transport in EVs (13–15). For neural circuit formation, neuronal exosomes promote neuronal precursor proliferation and cell differentiation both *in vivo* and *in vitro*. The EVs derived from human induced pluripotent stem cells (iPSC) are enriched in proteins related to neurodevelopmental functions including neuritogenesis and morphology of the nervous system. These functions are impaired due to the loss of MECP2, which also alters synaptic density and neuronal firing capabilities (16). In the subventricular zone, the neural stem cells release vesicles that function as morphogens by decreasing the number of microglial processes to induce its traditional stellate morphology. In response, microglia can reduce the proliferation of neural precursors through cytokine release (17). It appears that EV-mediated communication is an active system that continues into adulthood. In ultrastructural studies, the presence of vesicles in the intercellular space in various brain structures is notorious, which suggests the existence of communication axes between the different cell types in the brain: neurons, astrocytes, microglia, and endothelial and epithelial cells (18, 19).

At a neuronal level, synaptic maturation and remodeling are influenced by the molecular cargo of EVs. For example, the degradation of the post-synaptic density protein-95 is promoted by the delivery of Proline-Rich 7, which results in the elimination of excitatory synapses in rat hippocampal neurons (15). The release of EVs from the presynaptic terminal is activity dependent and promotes the delivery of molecules to the post-synapsis such as Synaptotagmin-4, which, by retrograde signaling, promotes the maturation of the presynaptic terminal at the neuromuscular junction of Drosophila (20, 21). Likewise, synaptic plasticity is influenced by EV-mediated transfer of Arc1 mRNA to the post-synaptic region (22, 23). While epileptic status selectively modifies the miRNA content of hippocampus-derived EVs, it does not modify the process of miRNA editing that occurs inside the vesicle, which modulates target recognition (24). These findings suggest that the information contained in the EVs add an extra layer of modulation for normal synaptic functioning, which can be bidirectional among neurons.

Regarding the communication between different brain cell types, there is evidence that EVs are targeted to specific cell types. Selective expression of CD63 coupled to GFP in neurons has been achieved in rodent models. Neuron-derived EVs containing GFP are incorporated preferentially by astrocytes and also in a minor proportion by certain neurons, both *in vivo* and *in vitro* experiments (25, 26). These vesicles are mainly secreted at the neuronal soma and dendrites, and they transfer the miR-124-3p, which in turn increases protein levels of the glutamate transporter GLT1 in astrocytes (26). Astrocyte-derived EVs seem to be directed mainly toward neurons to modulate a variety of functions (27). The effect of astrocyte-derived EVs depends on the brain microenvironment, being beneficial when there are trophic stimuli or neurotoxic when there are pro-inflammatory conditions. After incubating astrocytes with ATP, the EVs are enriched with proteins related to synaptogenesis (NETO1) and neurite growth (RPL10). In contrast, astrocyte stimulation with IL-1β or TNF-α enriches proteins and miRNAs associated with reduced growth and network neuronal activity (28, 29). In addition, the activation of astrocytes by IL-1β is associated with the production of vesicles that are released into peripheral circulation and that promote migration of leukocytes into the brain (30). In the case of microglia, an interaction with neurons through EVs has also been documented. The incubation of the MG6 microglial cell line with EVs from depolarized neurons promotes the degeneration of neurites from PC12 cells (31). A deleterious effect is also observed with the EVs derived from microglia BV2 cells after the activation of the metabotropic glutamate receptor 5, which is involved in neuroinflammation (32). LPS increases the levels of TNF-α and IL-6 in microglia-derived EVs and promotes the loading of proteins related to transcription and protein translation (33). Further research is needed to determine the role of microglia-derived EVs in normal conditions and after traumatic brain injury, which induces changes in miRNA cargo that have been related to anti-inflammatory effect on neurons (34).

Endothelial cells constitute another important participant of the intercellular communication in the brain. The survival and proliferation of oligodendrocyte precursor cells are promoted by EVs derived from microvascular endothelial cells (35). Likewise, endothelial cell-derived EVs inhibit apoptosis of neurons deprived of oxygen and glucose through the transfer of miR-1290 (36). Endothelial cells can also be influenced by astrocyte-derived vesicles that regulate the expression of tight-junction proteins (claudin-5, occluding, ZO-1) to maintain the integrity of the blood–brain barrier (37). After TNF-α exposure, the protein related to TNF signaling, immune response, and mitochondrial proteins are enriched in the EVs derived from endothelial cells (38). These findings indicate that EV secretion by endothelial cells has a role in the activation of an inflammatory systemic response. Indeed, a focal brain lesion induced by a microinjection of IL-1β in the brain parenchyma increases the circulating EVs from endothelial cells and induces the release of acute-phase response proteins by the liver (39). Conversely, the brain can be exposed to signals from the periphery. Breast cancer-derived EVs can cross the blood–brain barrier through a transcellular mechanism that involves the downregulation of Rab7 to switch the endocytic pathway to a recycling mode (40). The choroid plexus epithelium is another sensor of peripheral conditions that can modify brain cell physiology. After LPS injection in mice, choroid plexus epithelial cells release EVs into the CSF containing inflammatory miRNAs (miR-1a, miR-9, miR-146a, and miR155) that eventually activate an inflammatory response in astrocytes and microglia (19).

Because CSF contains a mixture of molecules originating from different cells in the brain, it has been used to monitor the physiological status of the CNS. In relation to EVs, there is evidence showing that the isolation of vesicles from CSF could be informative about the progression of neurological diseases. Recently, the analysis of patient-derived glioblastoma cells revealed that a subpopulation of EVs is mainly enriched in mRNA and non-vesicular ribonucleoprotein complexes are enriched in tRNAs and Y RNA fragments (41, 42). These changes can be detected by the analysis of EVs in CSF samples and help to provide a molecular diagnosis (43). The principle of monitoring pathological events in the brain is reinforced by the fact that EVs derived from peripheral blood reflect the presence of biomarkers of the developing tumor in the brain and its levels correlate with cell invasiveness (41, 42, 44). Similarly, the protein cargo of EVs from blood and CSF samples reflects early events of neurodegeneration related to mild traumatic brain injury and cognitive impairment (45–47). These proteomic profiling studies indicate alterations in the endosomal and lysosomal functions which are key processes underlying NDs. A possible role of EVs in the brain is to discard an overload of mis-folded proteins to maintain synaptic homeostasis (48, 49).

## Overview of the Role of EVs in Development and Propagation of NDS

Recently, it has been suggested that the classification of EVs in exosomes and microvesicles is an oversimplification of the heterogenous mixture of EVs that originate from different intracellular pathways (50, 51). While the exact composition and function of each EV subtype is still not well-defined, the current available methods for its molecular characterization indicate that a set of molecules associated with the development of NDs can be enriched or depleted under certain conditions. These changes may result from an alteration in the biogenesis of EVs or from a neuronal response to eliminate protein aggregates to counteract impairments in lysosomal and autophagy functions. In agreement with this notion, it has been observed that in several models of neurodegeneration, there are morphological alterations in the endolysosomal system, including larger multivesicular bodies (MVBs) with more intraluminal vesicles (52–54). In addition, neurodegeneration is related to changes in the expression of endolysosomal markers (EEA1, Rab5B, Rab7, CD63, TSG101, Rab35, LAMP1, and CTSD) and lipid metabolism (sphingolipids and lysobisphosphatidic acid) (49, 54, 55). Neurons with a reduced capacity of protein and organelle degradation are prone to accumulate protein aggregates, which apparently are propagated through EV release (Figure 1B).

Histological, biochemical, and proteomic analysis of EVs in different research models and patient samples have shown that Aβ and Tau in AD, and α-syn in PD are associated with EVs (56). In human brain sections from AD patients, the exosome marker flotillin-1 colocalizes with oligomers of Aβ (57). The injection of EVs isolated from familial AD patient-derived iPSCs in mouse hippocampus was sufficient to induce Tau phosphorylation in different regions of the hippocampus after 5 weeks (58). The Swedish mutation in the amyloid precursor protein (APP), which increases abnormal cleavage of cellular APP, favors the loading of Aβ40, Aβ42, and APP C-terminal fragments (CTFs) into EVs that are incorporated mainly by neurons (59). In an elegant study, it was shown that the disruption of the PI3K/Vps34 signaling pathway is involved in the secretion of APP-CTFs by causing a dysregulation of lipid metabolism that induces damage to endolysosomal membranes (49). A reduced EV release has been observed in a humanized APOE4 mouse model. The presence of the risk allele of apolipoprotein E is associated with decreased levels of Tsg101 and Rab35 (55). These findings are of great relevance considering that genetic risk factors of NDs usually involve proteins of endosomal and lysosomal trafficking. A compromised ability to handle cellular waste by the disturbance of endolysosomal trafficking can be reflected in the molecular content of EVs.

Similar evidence has been described for PD in which the dysfunction of key elements of lysosomal and endosomal pathways has been associated with the development of the disease (7). An analysis of PD human brain extracts showed that α-syn accumulation is associated with decreased levels of the lysosomal protein LAMP2 (60). For instance, the loss of the mitochondrial protein Parkin (PARK2) decreased the endosomal tubulation and increased the expression of late endosome markers such as CD63 and the number of intraluminal vesicles in MVBs (61). The overexpression of the lysosomal protein ATP13A2 (PARK9) increases its presence in MVBs and promotes the secretion of α-syn in EVs (62). Interestingly, the inhibition of the enzyme neutral sphingomyelinase (nSMase2), involved in the biogenesis of intraluminal vesicles, decreased the transfer of oligomeric aggregates of α-syn in co-culture experiments (63). The leucine-rich repeat kinase 2 (LRRK2) is naturally found in EVs from kidney, immune, and brain cells, and it is proposed to have a key role in autophagocytic pathway (64, 65). Levels of phosphorylated LRRK2 at residue S1292 in CSF and urine correlate with severity of the disease and help to predict mutation carriers (66). Overall, an early event in protein aggregation seems to involve components of the endosome and lysosome compartments.

## EV Proteins as Biomarkers in PD

PD is characterized by non-motor and motor alterations that are associated with the progressive loss of dopaminergic neurons in the substantia nigra pars compacta. A well-established histological feature is the presence of cytoplasmic inclusions known as Lewy bodies (LBs) that are composed of α-syn aggregates and sequestered organelles including mitochondria, endosomes, and lysosomes (67). Growing evidence indicates that α-syn pathology initiates in the periphery even earlier than in the central nervous system (68, 69). The mechanisms considered for α-syn pathology spreading include axonal transport, fluid phase, prion-like process, and EV-mediated transmission (70). Considering that EVs reflect cellular changes that occur in response to pathological conditions, it is conceivable that they are an effective source of biomarkers for both peripheral and central events of neurodegeneration.

Among the intracellular mechanisms proposed to explain PD pathogenesis, included is the disturbance of the interactions between mitochondria, lysosomes, and endoplasmic reticulum, which results in the accumulation of protein aggregates and cellular toxicity. The deletion of mitochondrial proteins (AIF, OPA, and PINK1) or inhibition of mitochondrial complex I with rotenone cause the appearance of lysosomal vacuoles, increase lysosomal permeability, and alter protein degradation (71, 72). The absence of PINK1 and Parkin induces the formation of mitochondrial-derived vesicles that can be shuttled to late endosomes and secreted to extracellular space (73). Similarly, the inhibition of the maturation of autophagosomes with bafilomycin promotes their fusion with multivesicular bodies and favors the release of α-syn through EVs (74). Altogether, these data indicate that dysfunction of organelle crosstalk not only creates a microenvironment for protein aggregation but also alters the dynamics of protein cargo that is packaged into EVs.

The protein α-syn is expressed in the brain and is present in an oligomeric or aggregated state in body fluids, CSF, and plasma (75). The levels of α-syn in EVs from CSF allow the discrimination of patients with PD and dementia with LBs from other neurological conditions and healthy controls (76). To achieve a better resolution, EVs from the central nervous system have been isolated by immunocapture using an anti-L1CAM antibody, which recognizes a cell adhesion marker enriched in neurons (77–79). The amount of neuron-derived EVs and α-syn levels in these EVs is higher in plasma of PD patients than in plasma of healthy controls (77, 79). A relevant point to mention is that measurement of α-syn in EVs from a specific cell population could provide a more consistent assessment of the quantity of a biomarker at different stages and types of NDs than measurements directly from total CSF or plasma. A cross-sectional study showed that α-syn in neuronal EVs is increased by two-fold in prodromal and clinical PD in comparison to multiple system atrophy, controls, or other NDs (80). Combined evaluation of α-syn and clusterin in neuron-derived EVs improves the differential diagnosis to predict PD from non-α-syn proteinopathies (80). Furthermore, an increased proportion of oligodendrocyte-derived EVs and astrocyte-derived EVs in relation to neuron-derived EVs in plasma shows a significant correlation with the Unified Parkinson's Disease Rating (UPDRS) part III scores in the patients with PD (79). Taken together, these data suggest that EVs derived from the central nervous system would be a helpful strategy with elevated specificity and sensitivity to differentiate and monitor the progression of PD.

The presence of relevant biomarkers in EVs has also been studied in other biofluids. In a small sample of Korean PD patients, α-syn was not detected in urine EVs (81). In contrast, the levels of α-syn oligomers and the ratio of α-syn oligomers to total α-syn is increased in salivary EVs isolated from PD patients (82). Additionally, the concentration of salivary EVs of neuronal origin is higher in PD patients than in control individuals, as well as the levels of phosphorylated α-syn (83). Tear fluid is also accessible and mirrors the pathophysiological changes in systemic and ocular diseases. A mass spectrometry analysis of tear samples in a small cohort of PD patients revealed that almost half of the deregulated proteins in PD are related to neuronal functions. Remarkably, DJ-1 levels are increased in PD tear samples vs. control tears (84). Dysregulation of core networks of proteins involved in lipid metabolism, oxidative stress, vesicle secretion, and immune response supports the participation of these mechanisms in PD pathogenesis (84). Further studies, including proteomic studies of tear fluid EVs in PD, are needed to deeper evaluate them as a feasible biomarker source for PD.

Regarding the observation of an altered EV content and secretion in PD patients, it can be correlated to impaired lysosomal function. Decreased glucocerebrosidase 1 (GBA-1) enzymatic activity in the brain is a common trait in PD. Dysfunction of the endocytic pathway through the inhibition of GBA-1 results in increased release of brain EVs containing α-syn oligomers (85, 86). The increased production of EV associated with α-syn contributes to accelerate protein aggregation in receptor cells *in vivo* (85). GBA1-associated neurodegeneration in PD is associated with impairment of the autophagic–lysosomal pathway and mitochondrial dysfunction, possibly attributable to the accumulation of dysfunctional mitochondria as a consequence of defective mitophagy (87). Moreover, α-syn aggregation can be involved in the dysregulation of autophagic and endolysosomal pathways and is also associated with mitochondrial dysfunction (88).

Mutations in the genes encoding for LRRK2 (PARK8) and DJ-1 have been associated with PD. The mutations in the kinase LRRK2 account for 40% of the familial cases and its loss of function alters the endolysosomal pathway. The most prevalent mutation LRRK2-G2019S interferes with the mitochondrial fission through increasing the expression of the factor DLP1 (89). Regarding the possible role of these proteins as biomarkers, it is known that the levels of DJ-1 and LRRK2 in urine-derived EVs differ between sexes (81). Notably, DJ-1 levels increase with age in PD male patients (81). A high ratio of Ser(P)-1292 LRRK2 to total LRRK2 in urine EVs seems to be useful in discriminating between LRRK2 mutation carriers and non-carriers with or without PD. Moreover, LRRK2 mutation carriers with PD have a higher ratio than control individuals (66). Also, Ser(P)-1292 LRRK2 levels correlate with the severity of cognitive impairment and difficulty in accomplishing activities of daily living (90). While its exact function in PD pathogenesis is not fully understood, DJ-1 may act as an antioxidant and chaperone and play a role in mitochondrial homeostasis, possibly involving the PINK1/Parkin pathway. The levels of DJ-1 in EVs derived from CNS and the ratio of EV DJ-1 to total DJ-1 are substantially higher in the plasma of PD patients compared to healthy controls (91). These data show promising results regarding the quantification of LRRK2 and DJ-1 in EVs and considering post-translational modifications could increase the accuracy for PD diagnosis and prognosis.

Biomarker discovery can be accelerated by the characterization of EVs using mass spectrometric analysis for the high-throughput identification of dysregulated proteins in NDs. Despite the challenges of EV isolation from a reduced sample volume and the purity of EV preparations, shotgun proteomic profiling experiments have started to reveal the families of molecules that are associated with EVs. A total of 1,033 proteins were identified in a proteomic analysis of EVs isolated from sera of healthy controls and PD patients in early stages of the disease. Of these, 21 proteins were upregulated and 2 were downregulated in PD samples, while the quantity of vesicles and the amount of classical exosome markers such as flotillin were similar (92). Among the most abundant proteins are the vacuolar protein sorting-associated protein 13D, peroxiredoxin-2, S100A8, cytochrome b-245 heavy chain, and syntenin 1 (92). A more refined analysis using neural immunocaptured EVs from serum identified 429 proteins samples from controls and patients with mild (Hoehn and Yahr score <3) and severe PD (Hoehn and Yahr score > 3) (93). The upregulated proteins in mild and severe cases include the clusterin, complement C1r subcomponent, afamin, apolipoprotein D, gelsolin, and pigmented epithelium-derived factor (PEDF). The down-regulated proteins in serum-derived EVs comprise human neuroblastoma cDNA clone CS0DD006YL02, complement C1q subcomponent, myosin-reactive immunoglobulin, Ig kappa chain, and Ig mu chain (93). In contrast, the proteomic characterization of plasma-derived EVs isolated by size exclusion chromatography showed that the levels of clusterin and C1r subcomponent were decreased in PD patients compared to control individuals (94). These disparate results highlight the importance of the isolation method and the need to determine whether differentially abundant proteins correspond to vesicular or extravesicular material.

Recently, a strategy to define the origin of EV subpopulations in blood was generated through the phenotyping of EVs by flow cytometry. The mean number of erythrocyte-derived EVs (CD235a^+^) correlated with the UPDRS scores for different disease stages. The proteomic analysis of EVs from erythrocytes revealed that 8 proteins of a total of 818 identified proteins showed significantly different levels as a function of PD stage (95). The protein markers that were highly expressed in controls include axin interactor dorsalization-associated protein (AIDA), alpha/beta hydrolase domain-containing protein 14Bv (ABHD14B), and glutamine-dependent NAD^+^ synthetase (NADSYN1), while proteins highly expressed in mild PD were quinoid dihydropteridine reductase (QDPR), alcohol dehydrogenase NADP^+^ (AKR1A1), and cannabinoid receptor-interacting protein 1 (CNRIP1). In contrast, the ubiquitin carboxyl-terminal hydrolase 24 (USP24) and ATP synthase subunit alpha mitochondrial (ATP5A1) were increased in moderate PD cases (Hoehn and Yahr score 2–2.5) (95). Future studies should explore differential protein signatures in other blood cell types such as immune cells, considering the relevance of persistent inflammation as a risk factor for developing PD.

The isolation of EVs from plasma, serum, and CSF samples represents several limitations including the limited volume obtained per patient and the technical procedures for its obtention. Conversely, urine samples can be used to maximize the yield of EVs and still capture the pathological processes occurring in different parts of the body. Consistent with this notion, a quantitative analysis of urinary EVs from a cohort of PD patients revealed that enriched proteins are related to neurological disorders including Parkinson's, Huntington's, and Alzheimer's diseases (96). Among the enriched endolysosomal proteins related to NDs, synaptosomal-associated protein 23 (SNAP23) and calbindin were particularly prominent in PD cases. The measurement of these proteins have a prediction success in a range of 76–86% for disease diagnosis in two independent cohorts (96). Moreover, this study demonstrated that EV-related proteins show low interindividual variability and can be tracked over time. Overall, these findings open the possibility of monitoring long term the abundance of proteins associated with neurological disorders and how their levels are modified in response to treatments.

Mitochondrial dysfunction causes oxidative stress favoring aberrant protein folding and protein accumulation (i.e., Aβ, huntingtin, Tau, and α-syn). Furthermore, mitochondrial damage and impairment of the removal of damaged mitochondria (mitophagy) have been proposed as a central event in the pathogenesis of PD. The analysis of circulant mitochondrial-derived vesicles can be promising for the identification of early biomarkers of PD, considering that it is a cellular mechanism of antigen presentation in inflammatory conditions (73). A recent characterization of small EVs from the serum of PD patients identified a reduction in the levels of mitochondrial markers such as adenosine triphosphate 5A (ATP5A), NADH: ubiquinone oxidoreductase subunit S3 (NDUFS3), and succinate dehydrogenase complex iron sulfur subunit B (SDHB), and of the levels of the tetraspanins CD9 and CD63 (97). A more precise protein signature of mitochondrial-derived vesicles should be addressed in future studies including genetic models of PD and patient samples. A better characterization of the temporal dynamics of mitochondrial markers in circulation would help to understand the interconnection with the endolysosomal system.

The studies described above account for the most recent advances in the identification of prospective protein biomarkers contained in EVs. However, some points need to be resolved before a valid biomarker profile based on EV characterization can be efficiently used for the diagnosis and prognosis of PD. Ideally, an exhaustive phenotyping of EV subtypes could improve the detection of altered proteins related to neurodegeneration. Moreover, a multicenter study that guarantees the standardization of the protocols used for EV isolation and proteomic analysis would benefit the phase of validation of biomarkers in large size cohorts. Finally, as the familial forms of PD are associated with endolysosomal or mitochondrial defects, a targeted analysis of proteins associated with these cellular pathways in the EVs derived from body fluids of PD patients would be of great value in understanding the evolution of the disease and determining its relation to pharmacological treatments.

## EVs Proteins as Biomarkers in AD

The histopathological hallmarks of AD are the presence of extracellular amyloid-β (Aβ) plaques and intracellular neurofibrillary tangles composed of hyperphosphorylated Tau protein. The exact mechanism that provokes this malignant protein aggregation is not fully understood. In the development of AD, EVs show a multifaceted role. Several studies have demonstrated a deleterious effect of EVs as carriers of pathogenic proteins that contribute to their spreading across the brain. However, others state that it is possible that EVs have a neuroprotective role by buffering the formation of toxic protein aggregates. The specific function of brain EVs seems to depend on their cellular origin and on the inflammatory status of the brain.

Neuroblastoma N2a-derived EVs act as scavengers of neurotoxic forms of Aβ by trapping the peptides on its surface through glycosphingolipids. These Aβ-EV complexes can be internalized by microglia for degradation (98). Moreover, the intracerebral infusion of EVs isolated from neuroblastoma cells diminishes Aβ pathology and amyloid deposition in APP transgenic mice (99). These findings suggest the use of EVs as a novel therapeutic approach to preventing plaque deposition in AD. In support of this notion, it has been proposed that the administration of EVs from hypoxic mesenchymal stem cells (MSCs) improves the learning and memory capabilities of the APP/PS1 mice by restoring the synaptic function. The EVs from hypoxic MSCs reduce the glial activation through the increase of anti-inflammatory cytokines (IL-10, IL-4, IL-6, and VEGF) and the decrease of proinflammatory cytokines (TNF-α and IL-1β) concomitantly *via* a reduction in the activation of the STAT3 and NF-κB pathway (100, 101). The administration of MSC-EVs also influences presynaptic functions by the induction of long-term potentiation (cellular correlate of learning and memory) at Schaffer collateral to CA1 synapses, which improves cognitive behavior (102). MSC-EVs also protect neurons by reducing oxidative stress through the transfer of catalase and by promoting synapse integrity (101).

An additional beneficial effect of EVs has been postulated through the transport of the cellular prion protein (PrPC), which is a glycoprotein with high affinity for the oligomeric form of the Aβ42. The EVs purified from N2a, SHSY-5Y, and cortical neurons, highly enriched in PrPC, show a higher binding affinity for dimeric, pentameric, and oligomeric Aβ species (103, 104). The EV–PrPC complex plays a protective role in sequestering soluble oligomeric forms of Aβ, reducing its neurotoxic effects by accelerating its fibrillization (103). A similar mechanism could also occur *in vivo* as the intracerebroventricular injection of human neural EVs in the brain rat prevents the inhibition of long-term potentiation caused by Aβ oligomers (105).

The notion of a neurotoxic role of EVs has been based on data showing the release of pathological forms of Aβ in the extracellular space by microglial and neuronal cells. As suggested by studies in AD animal models, EVs can diffuse throughout the brain and play a role in the dynamics of amyloid deposition (106). After Aβ internalization into microglia, fibrils can be converted into an oligomeric toxic form and reintroduced into endosomal secretion pathway (107, 108). In neurons, the loss of endosomal sorting complexes required for transport (ESCRT) components or the ubiquitination factor E4B (UBE4B) increases the levels of Aβ42 in late endosomes and its secretion through EVs (109). Both *in vivo* and cell culture assays have demonstrated that the dysfunction of the enzymes ECE-1 and−2, located in MVBs, could lead to the accumulation of intraneuronal Aβ aggregates and their subsequent release through EVs (110). Thus, mutations or alterations in the molecular machinery involved in APP trafficking can favor the loading and secretion of EVs with toxic aggregates into the circulation. Interestingly, the measurement of Aβ peptides and Tau levels in plasma-derived EVs has shown promising results in designing a score to follow AD progression and to discriminate from other types of dementias. With a sensitivity of 96%, the levels of phospho-Tau (pTau)-T181, pTau-S396, and Aβ42 levels in neuronal EVs discriminate AD patients from match-case controls. Moreover, EV levels of Aβ42 are an indicator of AD progression in preclinical cases (111).

The molecular characterization of EVs indicates that there is a basic set of proteins with a complex dynamic overtime, consisting of subtle changes in protein concentration and post-translational modifications. Mild cognitive impairment has been associated with lower levels of total Tau and APP and a higher ratio of pTau-T181/total Tau in comparison to controls. AD can be discriminated from healthy individuals considering the ratio of pTau-T181/total Tau, and from mild cognitive impairment considering the pTau-T181/total Tau ratio and the APP levels in plasma-derived EVs. In comparison to controls, the APP levels are lower in mild cognitive impairment and in mild and moderate AD, while high levels of APP characterize severe AD (112). These data indicate that the protein signature of EVs changes gradually during the development of AD. Other research groups have explored neuronal-derived EVs from plasma, the expression of transcriptional factors involved in neuronal defense against diverse stresses on AD patients. EVs of AD patients contain low levels of the low-density lipoprotein receptor-related protein 6 (LRP6), heat-shock factor-1 (HSF1), and repressor element 1-silencing transcription factor (REST). These transcription factors are diminished 2–10 years before the clinical diagnosis of AD (113).

In AD, the increased production of EVs is associated with the progression of Tau pathology (111, 114). The inhibition of exosome synthesis in microglia significantly reduces Tau propagation *in vitro* and *in vivo* (115). It is possible that EVs can contain different Tau species, including monomers, oligomers, and aggregates. However, only the aggregated forms can promote the aggregation of new Tau molecules. The release of EVs containing Tau is promoted by neuronal activity and thus may contribute to the spreading of Tau pathology through trans-synaptic transmission (116). EVs from the Tg4510 mouse (carrying the P301L Tau mutation) contain high levels of Tau with an altered pattern of Tau-phosphorylation (AT8, AT100, and AT180) that promote the formation of Tau inclusions. The P301L Tau-containing EVs transport Tau seeds, which are able to induce the aggregation of endogenous Tau in recipient cells, supporting the active role of exogenous seeds in nucleating nascent Tau inclusion through vesicle transport (117). Genome-wide association studies suggest a strong connection of late-onset AD with Bridging INtegrator 1 (BIN1), which is involved in endosomal trafficking (118). In animal models and CSF of AD, BIN1 is related to Tau secretion *via* EVs and could contribute to Tau pathology by altering Tau clearance and promoting the release of Tau enriched EVs by microglia (119). The characterization of EVs from human iPSCs, CSF, and plasma shows that the main type of Tau inside of EVs is the full length form, suggesting a dysfunction of autophagy (120). The presence of P301L and V337M Tau mutations in iPSC dysregulates the EV proteome. Some of the proteins exclusively present in mutant Tau-EVs have been reported to participate in cellular processes related to synaptic dysfunction, memory loss, and neuropathology. Remarkably, mutant Tau-EVs contain ANP32A, which is an endogenous inhibitor of protein phosphatase-2A (PP2A) that in turn regulates the dephosphorylation of Tau (121). Therefore, common mutations related to AD pathogenesis that alter endolysosomal trafficking may result in a noxious molecular cargo in EVs that contributes to the spread of toxic protein aggregates.

One of the major genetic risks associated with the development of late-onset AD is the allele ε4 of the apolipoprotein E (APOE4). The expression of APOE4 is associated with decreased levels of EVs in the human brain and in APOE4 transgenic mice. The exosomal biogenesis pathway seems to be compromised by the presence of APOE4, as indicated by the downregulation of the transcription and translation of Tsg101 and Rab35 in the humanized APOE mice (55). Additional effects related to APOE4 include enlarged endosomes, cholesterol accumulation, and increased secretion of Aβ42 (55, 122). The APOE4 genotype may contribute to the disruption of endosomal–lysosomal system function, probably due to disturbances in lipid metabolism that increase neuronal vulnerability over time. The exact mechanism has not been resolved, but it could involve a vicious cycle between astrocytes and neurons that ends in an inability of Aβ42 clearance and altered EV molecular cargo. A mass spectrometry analysis of the EV content from a mixed co-culture of mouse primary astrocytes, neurons, and oligodendrocytes found that the exposure to Aβ42 protofibrils altered the levels of ApoE, 2′,3′-cyclic-nucleotide 3′-phosphodiesterase (CNPase), heavy chain 1, 60S ribosomal protein L4, and cytoplasmic dynein 1 heavy chain 1 proteins (123). Previously, the same group showed that the inability of astrocytes to clear Aβ leads to the increased release of truncated forms of Aβ in EVs, thus inducing neural apoptosis (124). In line with these studies, an altered EV generation has been proposed from mass spectrometry analysis of neocortical brain tissue samples from AD patients. A pathway enrichment analysis revealed changes in proteins related to exocytic and endocytic pathways. Interestingly, among the proteins with higher levels in AD compared to control include EV-related markers such as CD9, HSP72, PI42A, TALDO, and VAMP2 (125). EVs from CSF and plasma of AD patients and AD mouse models expressing a presenilin-1 mutation show an increased Aβ42/Aβ40 ratio and impair Ca2+ handling and mitochondrial function (126). These findings suggest that distorted intercellular communication may contribute to neuronal dysfunction and highlights the role of glia in maintaining a healthy neuronal state.

Recent evidence also indicates the potential role of EVs in the transport of APP and its catabolites (CTFs). Mouse N2a cells expressing the Swedish mutation of human APP show a differential loading of CTFs into EVs. The Swedish mutation enriches CTF-α and CTF-β, but not CTF-η, into a subset of EVs that lack CD63. This subpopulation of EVs carrying CTFs only bind to dendrites of neurons, while CD63^+^ EVs are targeted to both neurons and glial cells (59). These changes in composition and cellular selectivity may result from an impairment of the endolysosomal function that modifies lipid composition, membrane proteins type, and protein glycosylation. The chemical inhibition or genetic ablation of the kinase Vps34 results in endosomal abnormalities and autophagic blockage in primary cortical neurons and N2a cells (49). The EVs collected from these cultures are enriched in CTFs, in cholesterol and sphingolipid subclasses (dihydrosphingomyelin, monohexosylceramide, and lactosylceramide), and in the phospholipid bis(monoacylglycerol)phosphate. Furthermore, the global protein glycosylation is modified in specific brain regions of AD patients, and some of these changes are reflected in serum samples (127). An abnormal protein glycosylation pattern has been associated not only with AD development, but also with a differential cellular uptake of EVs (128–130). The fact that APP-CTFs and Aβ are selectively sorted into a subpopulation of EVs and are specifically endocytosed by neurons suggests the mechanism in which EVs contribute to the spread of pathological fragments throughout the brain. In agreement with this notion, EVs isolated from human iPSC-derived neuronal cultures harboring the A246E presenilin-1 mutation can induce Tau hyperphosphorylation. Although the mechanism remains unknown, the injection of these EVs into the CA1 region of the mouse hippocampus generates Tau aggregation in the hippocampus after 5 weeks (58, 131). Likewise, EVs from mice containing Tau with the familial dementia mutation P301L accelerate Tau phosphorylation and soluble aggregate formation *in vivo* (132). These findings support the role of EVs in the spreading of Tau pathology in AD, but long-term studies are required to determine the intracellular mechanism by which EVs induce Tau phosphorylation and whether there is a direct participation in neurofibrillary tangle formation.

A dysfunctional endosomal system is an early feature of neurons of individuals with Down syndrome (DS), which includes larger endosomes and an accumulation of APP metabolites in endosomal vesicles. EVs isolated from the brains of DS patients, from the Ts2Cje mouse model of DS (Ts[Rb(12.17(16))]2Cje), and from human DS fibroblasts are enriched in APP-CTFs. The Ts2Cje model revealed that APP-CTFs levels in brain EVs increase in an age-dependent manner considering a period of up to 24 months of age (133). An electron microscopy study of the neuronal endosomal system of Ts2Cje mice shows that MVBs are larger, more abundant, and contain a higher number of intraluminal vesicles (ILVs) compared to controls. The biochemical analysis of Ts2Cje brain endosomes revealed an enhanced content of Rab5b, Rab7, CD63, Tsg101, and Rab35, suggesting an alteration in the biogenesis and secretion of EVs (54, 134). The up-regulation of the endosomal–EV pathway could be a homeostatic mechanism to improve endosomal dysregulation in NDs. In this regard, the brain-derived EVs from 3xTgAD mouse (APP Swedish mutation, Tau P301L, and Presenilin 1 M146V) or from neuroblastoma cells show an accumulation of APP-CTFs induced by the inhibition of the γ-secretase, a molecular complex involved in Aβ peptide generation. This seems to be a consequence of a defective retrograde transport of APP-CTFs from the endosomal compartments to the trans-Golgi network. The monomeric forms of APP-CTFs were mainly localized in the trans-Golgi network, whereas oligomeric forms were confined to endosomes and lysosomes, explaining the selective recovery of APP-CTFs in EVs (135).

The role of brain insulin resistance in the development of AD has been widely documented. Thus, the level of phosphorylation of the adaptor insulin receptor substrate (IRS)-1, a protein associated with the insulin signaling status, has been considered as a biomarker of cognitive changes. In a cohort of AD and diabetes mellitus, the ratio of phospho-serine 312-IRS-1 to pan-phospho-tyrosine-IRS-1 in neuronal EVs from plasma was found to discriminate AD cases from diabetic and control individuals. This insulin resistance index is higher in AD than in diabetic patients (136). Interestingly, the abnormal phosphorylation of IRS-1 could predict the development of AD up to 10 years before clinical manifestations and correlates with cognitive performance especially in APOE4 non-carriers (137). Moreover, the pS312-IRS-1/p-tyrosine-IRS-1 ratio in neuronal EVs has also been associated with the degree of brain atrophy in AD patients due to the volume of brain regions showing the presence of pS312-IRS-1, as assessed by magnetic resonance image (138).

Vascular pathology is considered an early event of AD pathogenesis. The reduction of blood flow affects the content of brain-derived EVs from mice subjected to bilateral common carotid stenosis. A mass spectrometry analysis of these EVs revealed the appearance of proteins implicated in neuroprotection (glutathione peroxidase and serine protease inhibitor A3), hypoxia (agrin and myosin light chain kinase, and EGF-containing fibulin-like protein), angiogenesis (angiomotin), and AD pathogenesis (sortilin-related receptor and PACS2). Similar groups of proteins are upregulated in EVs derived from brain tissue of preclinical AD and AD with cerebrovascular disease, and in EVs derived from the serum of mice subjected to blood flow reduction to the brain (139). These findings reinforce the notion that early brain damage could be monitored in the peripheral fluids, as has been recently documented in an analysis for the identification of markers for neurocognitive impairment. The proteome of CSF -derived EVs from HIV-positive individuals with neurocognitive disorders showed an enrichment of exosomal markers like Alix, Syntenin, tetraspanins, ARF, Rab proteins, and heat-shock proteins, as well as proteins related to synapses (NPTN, NRXNs, NPTXs, and SYN1), immune/inflammatory response (ANXA, CRP, DPYSL2, ENO1, EZR, and TIMP), stress response (GST, HSPs, PARK7, PRDX, SNCA, and SNCB), mitochondrial functions (ACOT, DNM1L, DNPEP, GLUD1, RAN, and VDAC), and the blood–brain barrier (GFAP, GLUL, AGRN, AQP1, AQP4, DAG1, FBLNs, and NIDs) (140). A chronic inflammatory environment has been considered a factor underlying early events of neurodegeneration associated with the progression of dementia. Advanced glycation end products such as N-(1-carboxymethyl)-l-lysine increase their formation during chronic inflammation, and their levels in serum-derived EVs can differentiate early to moderate AD (141).

The development of new and improved methodologies has enabled large-scale proteomics studies to characterize EVs obtained directly from brain tissue of animal models and AD patients (142, 143). This kind of study allows the analysis of the EV composition while preserving the tissue microenvironment and without physiological alterations associated with cell cultures. The proteomic analysis of the EVs isolated from frozen brains of mouse AD models have shown that their contents change along with the development of histopathological and behavioral hallmarks of AD. In a mouse containing mutations in the APP and PSEN1 genes (5xFAD), the EV proteome reflects complex changes in protein abundance that involve both the increase and the decrease of various components differentially at 2 and 6 months of age. For instance, at 2 months, the proteins neuromodulin (Gap43), microtubule-associated protein 2 (Map2), glia maturation factor beta (Gmfb), and oxidation resistance protein 1 (Oxr1) are enriched in the EVs of AD mice. However, at 6 months, these proteins are markedly decreased in the EVs of AD mice (143). A proteomic profiling of human brain-derived EVs suggested that EV biogenesis might be altered in preclinical AD as indicated by an increment of MHC class I levels. In addition, EVs of preclinical cases are enriched in proteins that indicate the activation of immune response (SSBP1, PAF, and CD90), a modulation of synaptic structure (NLGN3), and a dysregulation of mitophagy (GABARAP). The progression of AD is characterized by an alteration in lysosome dynamics (LAMP1), an upregulation of amyloid associated proteins (APP, PrP, and ENPP2), and an induction of neurite regeneration (GAP43) (144). A similar analysis of brain-derived EVs from diagnosed AD cases revealed an increase of Aβ42 and pS396-Tau levels in AD samples as determined by ELISA. The label-free proteomic comparison of these EVs identified a set of differentially expressed proteins in the samples from the brain of AD patients, including APOE, SNCA, ANXA5, MITCH2, GPM6A, VGF, and ACTZ. Interestingly, ANXA5 shows a positive correlation with Braak stages (145). As can be appreciated from these studies, there is not an exact match of the modifications of the EV cargo. Moreover, the central core of proteins remains constant between healthy individuals and patients. This highlights the importance of the protocols employed to isolate different subpopulations of EVs and the inclusion of quantitative approaches to detect subtle changes in the abundance of key proteins.

## Current Advances and Challenges in Proteomic Characterization of EVs

During the last two decades there have been numerous efforts directed to determine the protein composition of several biofluids in health and disease. One of the main challenges in the molecular characterization of any biofluid is that the difference of concentrations between the most and the least abundant proteins can cover more than 10 orders of magnitude (146, 147). Currently, the most common and efficient method for studying complex protein mixtures is mass spectrometry; however, the limit of detection of modern instrumentation is up to five orders of the proteome dynamic range (148). This introduces a bias where only abundant proteins are confidently detected, while low-abundant proteins escape identification. Over the past few years, several strategies including labeling techniques, fractionation protocols, acquisition methods, and bioinformatic pipelines have been implemented to increase the resolution of proteomic analysis and improve quantification of proteins in clinical samples. As a result of this refinement of the mass spectrometry analysis, the compendium of plasma proteins went from 280 identified proteins in 2002 to 3,509 proteins in 2017 (147, 149). Nevertheless, there are still limitations in finding consistent biomarkers, especially in early phases, for AD and PD. In this regard, EVs proteomics, especially from plasma, represents a promising strategy to enrich relevant molecules based on the fact that its cargo reflects the physiological state of a cell population. To further advance the discovery of biomarkers for NDs, there is a need for the standardization of protocols for EV isolation to assure reproducibility in the measurement of altered proteins.

Recent advances in the EV field have shown that there are diverse routes involved in their biogenesis, and potentially each vesicle subtype can indicate the dysfunction of a cellular process. A challenge remains in designing strategies to efficiently separate EV subtypes to identify pathogenic cargo. Most of the protocols available to isolate EVs rely on the size and density of vesicles. The proteomes of EVs from different cell sources and biofluids obtained by ultracentrifugation, precipitation, size exclusion chromatography, or ultrafiltration display components that are not necessarily EV components. The use of bottom-loaded density gradients has served to separate soluble components previously thought to be part of EV cargo such as histones, ribonucleoproteins, Argonautes, and major vault protein (51). Furthermore, the identification of proteins sensitive to trypsin digestion combined with a systems biology approach has helped to gain an insight into the real components of EVs, suggesting the presence of contaminants even after the combination of several methods of isolation (150). Particularly for CSF- and plasma-derived EVs, the elimination of non-vesicular components should be addressed in the future as a great proportion of the proteins identified in mass spectrometry studies include IgGs, coagulation factors, histones, and complement factors. The successful recovery of brain-derived EVs is one step forward in favor for the detection of low abundant proteins that could be relevant for biomarker discovery (77–79).

A general conclusion of the studies involving the proteomic analysis of EV samples from AD and PD patients is that there is a big overlap of proteins between healthy individuals and patients. Only a few dozens of proteins are exclusively found in the disease state, at least with the resolution of current methods. This highlights the importance in developing quantitative proteomic approaches with enough sensitivity that will not only register an alteration in the concentration of certain components but also allow for patient stratification (151, 152). A simple but powerful modification in the protocol of EV isolation accompanied by an exhaustive fractionation of the samples with nano-ultra-high-performance liquid chromatography has proved to impact the resolution of mass spectrometry analysis. Over 5,000 proteins related to the biogenesis and function of small vesicles were identified in an analysis of EV-derived from plasma obtained at low-speed centrifugation (20,000 × *g*) (153). These findings open the possibility to analyze other clinical samples with the same strategy and to focus on determining the tissue origin of EVs. As we detailed in this review, alterations in the phosphorylation and glycosylation of certain proteins is an aspect to be included in future mass spectrometry analysis due to its relevance in development of neurodegeneration. We envision that the study of post-translational modifications would accelerate the discovery of cellular pathways altered in the development of PD and AD.

## Conclusions and Perspectives

There is a growing interest in the proteomic analysis of EVs due to their potential to transport biomarkers indicating the development of various chronic and degenerative diseases. The recent technical advances in proteomics have accelerated the characterization of EVs, revealing that their cargo reflect the cellular changes that occur in physiological and pathological conditions. Recently, several research groups have begun to elucidate the role of EVs in the progression of AD and PD. At a cellular level, both diseases present alterations in the interaction between endosomes, lysosomes, and mitochondria, which are considered as early events of neurodegenerative processes. The dysfunction of these organelles affects the biogenesis and release of EVs by the cells in the brain. Interestingly, several genetic risk factors of NDs involve proteins implicated in the endosomal and lysosomal trafficking. The disturbance of the endolysosomal system alters normal intracellular trafficking and organelle turnover. These events are reflected in impared degradation of proteins and alterations in protein sorting (Figure 1B). Overall, the cell ability to handle cellular components and waste is compromised, altering the molecular content of EVs.

**Figure 2:**
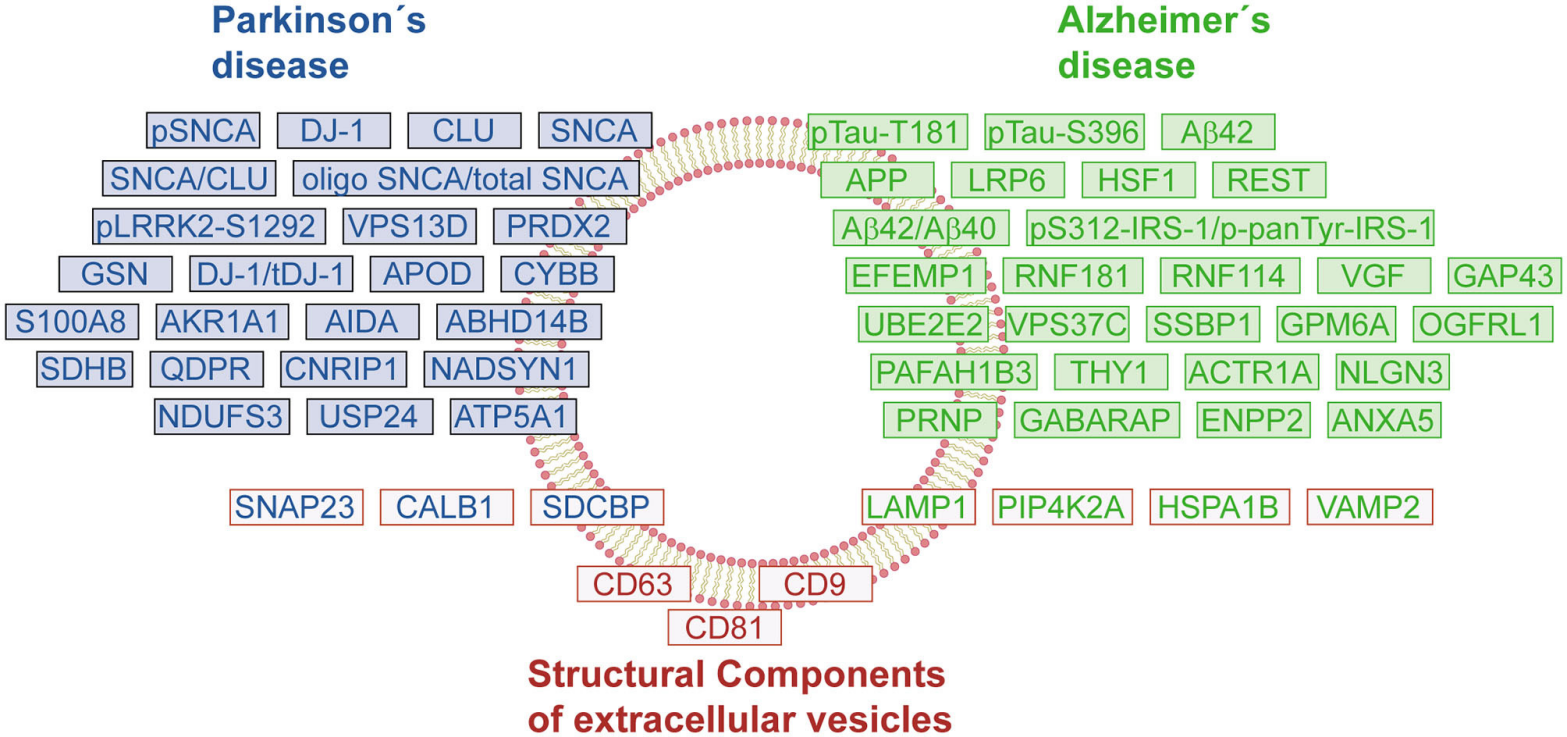
Protein signature of EVs in Parkinson's and Alzheimer's disease. The schematic representation shows a compilation of changes in the protein content of EVs under pathological conditions. The proteins/genes were classified by groups: Parkinson's disease (PD, blue boxes), Alzheimer's disease (AD, green boxes), and endolysosomal pathway-related proteins or structural components of EVs (red boxes). The color-coding of protein names follows the same classification criteria.

To further promote the use of EVs as biomarkers of NDs, it is necessary to develop standardized isolation protocols that ensure the quality and purity of the EV preparations. Moreover, considering that a central core of proteins remains constant between healthy and diseased individuals, the characterization of the different subpopulations of EVs is essential to identify those vesicles relevant for diagnosis purposes, and to follow the progression of NDs. During the discovery and verification phases, it will be key to correlate the EV cargo (protein, RNA, or metabolites) with relevant clinical data. The current discoveries concerning EV proteomes have already generated a list of interesting protein markers and post-translational modifications that should be corroborated in large cohorts (Figure 2). It is expected that in the following years, more candidates will be available that allow a differential diagnosis between different NDs. The EV proteins can also be useful biomarkers to follow the therapeutic response in drug trials (154). In the future, the development of new technology that provides enough sensitivity, wide dynamic range, and reliable quantification without an enrichment step of EVs would be desirable (155).

From a mechanistic point of view, a remaining challenge is to determine whether the reported changes in the content of EVs in AD and PD are a functional mechanism of communication or an indirect effect of the impairment of the management of cellular waste. This will help in the understanding of how the process of neurodegeneration starts and will give an insight into possible alternatives to halt the development of NDs. In this regard, it will be useful to perform cross-sectional studies to determine the molecular content of EVs at different ages and consider peripheral sources of EVs to evaluate its influence on the function of the nervous system. An omic approach considering the association of EV cargo with blood-relevant analytes (e.g., inflammatory, metabolic) combined with brain imaging will help to build a strong diagnostic strategy.

## Author Contributions

RQ-B and KH-O wrote the manuscript and EM-M conceived and wrote the manuscript. All authors contributed to the article and approved the submitted version.

## Conflict of Interest

The authors declare that the research was conducted in the absence of any commercial or financial relationships that could be construed as a potential conflict of interest.
